# A multi-level intervention to eliminate hepatitis C from the Region of Southern Denmark: the C-Free-South project

**DOI:** 10.1186/s12879-022-07196-7

**Published:** 2022-03-01

**Authors:** Sandra Dröse, Anne Lindebo Holm Øvrehus, Dorte Kinggaard Holm, Lone Wulff Madsen, Belinda Klemmensen Mössner, Jacob Søholm, Janne Fuglsang Hansen, Birgit Thorup Røge, Peer Brehm Christensen

**Affiliations:** 1 Department of Infectious Diseases, Odense University Hospital, J. B. Winsloews Vej 4, Indgang 18 Penthouse 2. sal, 5000 Odense C, Denmark; 2 Department of Clinical Research, Faculty of Health Sciences, University of Southern Denmark, Winsløwparken 19, 3. sal, 5000 Odense, Denmark; 3 Department of Clinical Immunology, Odense University Hospital, 29 J. B. Winsloews Vej 4, Indgang 8, Odense C, 5000 Odense, Denmark; 4 Department of Medicine, Lillebaelt Hospital, Sygehusvej 24, 6000 Kolding, Denmark

**Keywords:** Hepatitis C virus, Elimination, Epidemiology, Interventions, Testing, Treatment

## Abstract

Denmark has signed the WHO strategy to eliminate hepatitis C virus (HCV). In the absence of a national strategy for elimination, a local action plan was developed in the Region of Southern Denmark (RSD). The aim of the strategy is to diagnose 90% of HCV-infected persons and treat 80% of those diagnosed by 2025. The strategy was developed by reviewing Danish data on HCV epidemiology and drug use to identify key populations for screening, linkage to care, and treatment. Based on available published data from 2016, an estimated 3028 persons in the RSD were HCV-RNA positive (population prevalence 0.21%). Of these, 1002 were attending clinical care, 1299 were diagnosed but not in clinical care, and 727 were undiagnosed. Three different interventions targeting the HCV-infected population and two interventions for HCV surveillance are planned to achieve elimination. The “C-Free-South” strategy aims to eliminate HCV in our region by identifying (90%) and treating (80%) of infected persons by the end of 2025, 5 years earlier than the WHO elimination target date.

## Background

Hepatitis C virus (HCV) accounted for more than 71 million infections worldwide as of 2015, corresponding to a global prevalence of 1.0% [1–3]. Over a period of 20–30 years, 10–20% of the chronic hepatitis C infected will advance to cirrhosis, of whom 2–5% per year will develop liver failure or hepatocellular carcinoma (HCC) [3]. With the introduction of new direct-acting antivirals (DAAs), it has become possible to eliminate HCV and prevent development of cirrhosis and HCC. DAAs have caused a paradigm shift in hepatitis C treatment. DAA treatment is short and simple, has a cure rate of > 95% regardless of genotype, and has very few side effects compared with earlier interferon treatment [4]. The World Health Organization (WHO) assembly decided in 2016 to work to eliminate hepatitis C by 2030. The aim is to reduce new infections by 90% and hepatitis C related death by 65%. To achieve these targets it is necessary to identify 90% of infected persons and to start treatment in 80% [5]. It is important to improve established health care services, as the diagnosis and treatment uptake depend on them. This is best illustrated in the “cascade of care”, which describes a continuum containing an estimation of the HCV prevalence and the number of diagnosed persons and, of the latter, the numbers linked to care, treated, cured of HCV infection, and assessed post cure [6]. Monitoring all the steps in the cascade and organizing interventions both improves adherence to the pathway for the HCV infected and are necessary in achieving elimination. Many countries have signed up to eliminate HCV by 2030 and some have defined national or local strategies [7–11]. Hepatitis C demographics differ from country to country, and thus there is a need for various action plans with interventions tailored to each setting [5, 12]. In most of the Western world, people who inject drugs (PWID) drive the hepatitis C epidemic, and this vulnerable group poses specific problems at each step of the cascade of care [13]. Several studies have shown that re-infection does occur after treatment with DAA in this population at various rates (1–10 per 100 person years), depending on risk behavior [14–18]. Additionally, with the new treatment options, there is an obvious need to contact patients lost to follow-up and other known hepatitis C infected persons in the registers [9, 19–21]. Furthermore, studies from other countries on testing for hepatitis C in emergency departments (ERs) have shown that it can detect undiagnosed cases [22–24]. In a nationwide, population-based, cross-sectional study performed in Sweden in 2017, an HCV prevalence of 4.6% was found among persons with a psychiatric diagnosis [25]. It is assumed that there is a hidden group of HCV-infected persons who could be detected if screening tools were available and used when they visit the hospital for other reasons. Persons not in contact with the health system can be reached with a mobile clinic. There are examples of outreach mobile clinics providing hepatitis C screening and linkage to care and, in some cases, providing treatment [26–29]. In order to achieve the WHO goal of diagnosing 90% of infections, it is important to have a valid and updated estimate of HCV infections in every country [30, 31]. Recent data show that only 11 of 45 high-income countries are on track to achieve the elimination targets by 2030, and Denmark has been classified as a country not on track [32, 33]. Denmark has endorsed the WHO strategy to achieve hepatitis C elimination, and a national strategy to reach the targets is under development but not yet available. The country has 5.8 million inhabitants, with a low prevalence of hepatitis C of 0.21% in the general Danish population, and ~ 85% of hepatitis C infections are due to injection drug use [34–36]. In the absence of a national plan, our team decided to design an action plan aiming to eliminate HCV in the Region of Southern Denmark (RSD) by targeting multiple populations with a variety of interventions. The principal aim is to achieve the WHO goals of 90% of hepatitis C infected persons diagnosed and 80% treated in the RSD and to do so by 2025. The purpose of this article is to describe how we designed the micro-elimination strategy for the RSD based on available data.

### Study setting and population of the RSD

**Fig. 1:**
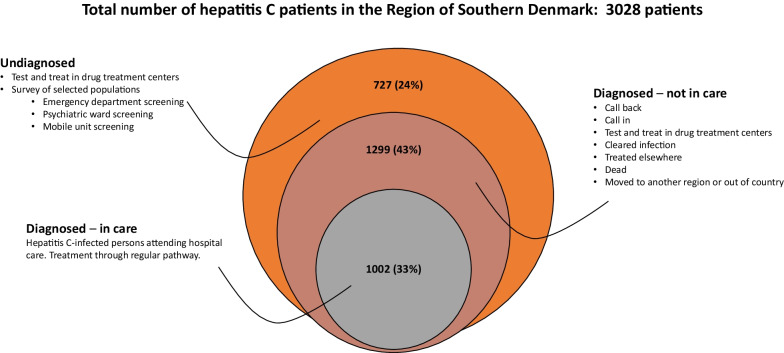
The estimated numbers of hepatitis C (HCV) infected individuals in the Region of Southern Denmark [34]

In the RSD, with a population of around 1.2 million inhabitants and an area of 12,260 km^2^, HCV treatment is provided by two clinical departments [37, 38]. Treatment of hepatitis C infections was restricted to patients with significant fibrosis until November 2018, but is now available to all HCV-infected patients [39, 40]. Tracking of patients in Denmark is facilitated by the use of a 10-digit personal identification number (PIN) used in all public systems and assigned to all permanent residents in Denmark. A hepatitis C survey of the general population in Denmark has never been performed, but a national estimate from 2016 was made based on registry data [34]. The infected population was primarily men born in the period 1950–1980, with current or former residence in larger cities [34, 41, 42]. In the RSD, the estimated total number of patients with hepatitis C infection was 3028 (95% CI 2044–5411). Of these, 1002 (33%) attended clinical care, 1299 (43%) were diagnosed but without contact with the health care system, and 727 (24%) were undiagnosed [34] (Fig. 1). Additional data sources were identified to estimate the sizes of populations at risk for undiagnosed or untreated infections [34, 43, 44]. On the basis of these data, a multi-level intervention program consisting of five interventions has been designed with the goal of achieving elimination. The regional strategy was launched in January 2019, shortly after treatment became available to all patients. An overview of the interventions is shown in Table 1.

**Table 1:** An overview of the five interventions in the Region of Southern Denmark under the C-Free-South Project to eliminate hepatitis C

	Name of intervention	Target population	Number of estimated HCV patients	Study type
Intervention 1	Test and treat in drug treatment centers and prisons	PWUD in DTCs in the Region of Southern Denmark: 15 centers with around 3000 persons enrolled	600	Health systems intervention study with register-based baseline and outcome evaluation
Intervention 2	Call back	Group 1) Call back: HCV infected and lost to follow-up, based on clinical records	100	EMR-based recall
	Call in	Group 2) Call in: HCV persons never engaged in HCV care	800	Clinical cohort study (part of a national study)
Intervention 3	Survey of selected populations	Emergency departments: Risk-based HCV screening	600	Prospective cohort study
		Psychiatric wards: Screening of all acutely HCV-infected persons by screening		Prospective cohort study
		Mobile unit outreach: HCV screening of persons utilizing shelters, cafés, and facilities targeting people with drug or alcohol misuse and/or psychiatric illnesses		Prospective cohort study
Intervention 4	A survey of drug-related death	PWID	–	Nationwide register study
Intervention 5	Re-infection and outcomes in patients treated for hepatitis C infection with recent injecting drug use or on opiate substitution therapy	PWID	–	Prospective multi-center cohort study

*DTC* drug treatment center, *EMR* electronic medical record, *HCV* hepatitis C infection, *PWID* people who inject drugs, *PWUD* people who use drugs, *RSD* Region of Southern Denmark

### Intervention 1: Test and treat in drug treatment centers and prisons

#### Current status

Testing and treatment for hepatitis C in the RSD was initiated in two drug treatment centers (DTCs) and one prison on Funen in 1996 [45–47]. A registry-based cohort study with people in drug use treatment based on 1996–2015 data found a prevalence of 62% HCV exposure and 33% HCV infection in people who reported ever injecting and who were alive at the end of 2014 [43]. The HCV test uptake in persons connected to six DTCs in the RSD was 52%, while the HCV test uptake in the remaining nine DTCs is unknown but assumed to be much lower [43]. According to national guidelines, yearly testing for hepatitis B and C is recommended for clients attending DTCs [48, 49].

#### Target population and potential barriers

The DTCs in the region provide care for an estimated 3000 people who use drugs (PWUD). Only 2 of 15 DTCs in the region have previously been engaged with all levels of the cascade of care, while others have implemented facilitated referral for treatment but have not engaged in other steps. Many centers have never been engaged in hepatitis C outreach care. In Denmark, only DTCs are allowed to provide opioid agonist therapy (OAT), mainly buprenorphine or methadone, and in two centers heroin-assisted therapy is offered. In addition, many centers provide needle-and-syringe programs (NSPs). A major obstacle in identifying HCV-infected persons in outreach settings is poor access to blood sampling and a lack of focus on HCV testing in many DTCs.

#### Interventional design and strategies to overcome barriers

A “test and treat model” will be sequentially implemented at all DTCs in the RSD, with annual follow-up testing after a sustained viral response is established. Each DTC starts with a test intervention phase initiated with the training of health and social workers in dried blood spot (DBS) testing and a general introduction to hepatitis. During the test intervention (months), the staff in the DTC will be encouraged to test all clients at the DTC, with the aim of testing more than 90% of PWUD. A treatment intervention will follow the test intervention. An outreach team doctor and/or nurse will visit the center equipped with a fibroscan™, a computer with access to patient electronic medical records (EMR), and equipment for venipuncture. After the assessment, patients will be offered treatment according to guidelines, and if it is accepted the DTC will dispense the drugs [50]. Treating as many people at the same time as possible in one DTC is prioritized in order to reduce re-infection. The baseline and post intervention test rates and HCV prevalence will be calculated from crosslinking the Registry of Drug Abusers Undergoing Treatment with the National Hepatitis Laboratory Register (DANVIR) [34]. Treatment uptake and outcome data will be generated by the regional clinical hepatitis database [51]. Testing by DBS has the advantage of very simple collection of blood samples that provide both HCV-RNA and anti-HCV, making it ideal for surveillance of both exposure and current (re-)infection. We have implemented testing by DBS at prisons in our region, with an 80% participation rate among prisoners [52, 53].

#### Expected effect of the intervention

We expect that 600 hepatitis C infections will be detected. Most of them will belong to the group of undiagnosed persons, but a significant proportion will belong to the group of diagnosed persons not in care, depending on the historic testing rates at each DTC.

### Intervention 2: Call back and call in: Contact with patients identified in national registers who have not entered care or were lost to follow-up

#### Current status

Due to the historical lack of treatment options, many patients dropped out of clinical care before unrestricted DAA treatment became available.

#### Target population and potential barriers

The population consists of two groups. Group 1 (call back) comprises hepatitis C infected patients who had been attending the hepatitis clinics but dropped out before being offered treatment. Group 2 (call in) consists of persons noted in public registers as having hepatitis C but who never attended clinical care [34].

#### Interventional design and strategies to overcome barriers

Both groups will be contacted twice with a letter, either electronic or mailed, or by phone, depending on local feasibility. If a patient does not respond, their general practitioner will be informed of the possibility of referring the patient. Patients who respond will be offered treatment through one of the two infectious disease departments in the region.

#### Expected effect of the intervention

The call-back group is estimated to comprise around 100 persons. The call-in group is estimated to comprise around 800, but with a large uncertainty of the validity of recorded data, which can be improved by EMR review. Based on the available literature, we expect 30–40% of notified patients to attend the clinics after two notifications.

### Intervention 3: Survey of selected populations

#### 3a. Emergency departments: Detecting hepatitis C infections by screening

#### Current status

The HCV status of patients referred to emergency departments has never been surveyed in Denmark. Studies from other countries have shown it could be feasible to screen this population. Therefore, we will examine HCV prevalence by screening patients in the emergency department in the hope of detecting an undiagnosed hepatitis C population.

#### Target population and potential barriers

Patients admitted to the ER for a medical reason will be asked for their current HCV status and risk factors. Drug-affected or unconscious patients will not be included in the study, and this could have an influence on the HCV prevalence.

#### Interventional design and strategies to overcome barriers

A 3-month prospective cohort study will determine the prevalence of diagnosed and undiagnosed cases of hepatitis C infections at Odense University Hospital, a large urban emergency department in Denmark, by using a risk-based, point-of-care screening strategy. Patients found to be HCV infected will be linked to care, and treatment will be initiated.

#### Expected effect of the intervention

We aim to include 500 patients in the pilot study. If our expected finding that 3% of subjects have chronic HCV infection is confirmed, the study will be rolled out to all emergency departments in the RSD.

#### 3b. Psychiatric wards: Testing and linkage to care for viral hepatitis in patients admitted for psychiatric care

#### Current status

A significant proportion of patients with mental illnesses have chronic hepatitis C [54]. There are no published studies on the prevalence of hepatitis C in people with psychiatric co-morbidities in Denmark. In the pre-DAA era when interferon was the standard treatment for HCV, people with a psychiatric diagnosis were rarely offered treatment because of its neuropsychiatric side effects. However, with the ease of DAA treatment and the presumed high HCV prevalence in this group, diagnosing and initiating hepatitis C care in psychiatric clinics is a quite obvious strategy to implement.

#### Target population and potential barriers

Subjects will include all patients admitted to the psychiatric department at Odense University Hospital or the psychiatric emergency ward at Vejle Hospital.

#### Interventional design and strategies to overcome barriers

For a period of 3 months, patients at the psychiatric department at Odense University Hospital will be screened for hepatitis B and C as part of the admission routine. All patients attending the psychiatric emergency ward at Vejle Hospital will be offered screening for hepatitis C with a point of care quickest for a period of 3 months. Hepatitis C infected individuals will be linked to HCV treatment at both locations.

#### Expected effect of the intervention

By screening patients for hepatitis C upon admission to psychiatric care, we estimate that 5% of those tested will be HCV infected and assume that 25% of these diagnoses will be new. A direct linkage to care and assessment for viral hepatitis will be facilitated. If deemed feasible and with an HCV prevalence > 2%, the screening will be extended to cover all psychiatric wards in the RDS.

#### 3c. The effect of a mobile outreach intervention: Hepatitis C test uptake, prevalence, and linkage to care among marginalized populations

#### Current status

A large part of the RSD’s population has limited access to low-threshold HCV tests. With a mobile clinic we will reach this population and estimate the prevalence of HCV in these untested parts of the RSD.

#### Target population and potential barriers

The mobile clinic will visit non-governmental organizations, targeting people in the RSD with drug or alcohol use with or without psychiatric diseases. Besides visiting shelters and street kitchens, the mobile unit will also visit music festivals and other gatherings with expected high prevalences of HCV infections.

#### Interventional design and strategies to overcome barriers

The mobile clinic will provide information and counseling about HCV infection and point-of-care anti-HCV and HCV-RNA testing (GeneXpert) by finger prick. In those found to be infected with HCV, liver stiffness measurement and treatment will be initiated. The mobile unit will function as an outreach clinic where patients will be affiliated to their local hospital-based outpatient clinic in their area of residence.

#### Expected effect of the intervention

We aim to screen 1500–2000 patients and to identify 50–100 HCV patients who will be offered treatment during a 2-year period.

### Intervention 4: A survey on drug-related death

#### Current status

To monitor the effects of the different interventions described above and to identify when the WHO targets have been met, it will be necessary to establish active surveillance. In a national prospective cohort study of drug-related deaths in Denmark spanning 2004 to 2009, post-mortem blood samples were tested for anti-HIV, anti-HCV, and anti-HBc [55, 56]. In 2004, among 233 drug-related deaths, the prevalences of these antibodies were 4%, 51%, and 35%, respectively.

#### Target population and potential barriers

For monitoring hepatitis C infections among PWID not attending DTCs, we will resume HCV prevalence surveillance of drug-related deaths in Denmark.

#### Interventional design and strategies to overcome barriers

The study will be performed as a register-based study where drug-related deaths will be extracted from the national mortality register, and the percentage of those tested for hepatitis C and of those found HCV positive prior to death will be extracted from the national laboratory database. Due to changes in ethics legislation, it will not be feasible to perform post-mortem HCV testing as was done in the previous study.

#### Expected effect of the intervention

We expect to find a decline in HCV infection in drug-related deaths in our region and we will be able to validate attainment of the WHO diagnostic target of 90%.

### Intervention 5: Re-infection and outcomes in patients treated for hepatitis C infection with recent injecting drug use or on opiate substitution therapy

#### Current status

People who inject drugs are a key population in the efforts to achieve reduction in new infections Re-infection implies gaps in prevention measures such as OAT and NSP and might identify potential actions for harm reduction. As of yet there are no Danish data on re-infection rates.

#### Target population and potential barriers

In Denmark, persons cured of HCV are discharged from care after cure, unless they have cirrhosis. Persons on OAT should be offered yearly surveillance testing according to national guidelines, but the uptake is presumed to be very low.

#### Interventional design and strategies to overcome barriers

A prospective cohort study at one of the largest DTCs in the RSD will offer a 3-year follow-up with evaluation every 6 months and subsequent retreatment for HCV infection if identified. In the remaining 14 DTCs, an annual retest will be ensured. These data will validate registry-based data on re-infections that might be flawed by insufficient test uptake.

#### Expected effect of the intervention

We expect to obtain reliable data on the re-infection rate among high-risk groups in a major city in Denmark.

## Conclusion

This paper is a recipe for the possible elimination of hepatitis C in a Danish region of 1.2 million inhabitants. The next years will show if this is feasible. The conditions in Denmark are favorable, with a low prevalence of hepatitis C, free access to DAAs, excellent registries, a good healthcare system, and dedicated people working in DTCs among patients with few resources. We hope to report the results of each of the interventions and how best to reach the WHO HCV elimination targets in Denmark.

## Data Availability

Data sharing is not applicable to this article, as no datasets were generated or analyzed during the current study.

## Abbreviations

DAA: Direct acting antivirals
DBS: Dried blood spot
DTC: Drug treatment center
EMR: Electronic medical records
HCC: Hepatocellular carcinoma
HCV: Hepatitis C virus
HIV: Human immunodeficiency virus
NSP: Needle-and-syringe program
OAT: Opioid agonist therapy
PIN: Personal identification number
PWID: People who inject drugs
PWUD: People who use drugs
RSD: Region of Southern Denmark
WHO: The World Health Organization
